# Differential roles of ARID1B in excitatory and inhibitory neural progenitors in the developing cortex

**DOI:** 10.1038/s41598-021-82974-y

**Published:** 2021-02-16

**Authors:** Jeffrey J. Moffat, Eui-Man Jung, Minhan Ka, Byeong Tak Jeon, Hyunkyoung Lee, Woo-Yang Kim

**Affiliations:** 1 Developmental Neuroscience, University of Nebraska Medical Center, Omaha, NE 68198 USA; 2 Department of Molecular Biology, Pusan National University, Busan, 46241 Republic of Korea; 3 Research Center for Substance Abuse Pharmacology, Korea Institute of Toxicology, Daejeon, 34114 Republic of Korea; 4 Department of Biological Sciences, Kent State University, Kent, OH 44242 USA; 5 Present Address: Department of Neurology, University of California San Francisco, San Francisco, CA 94153 USA

**Keywords:** Neuroscience, Development of the nervous system, Neural progenitors, Neuronal development

## Abstract

Genetic evidence indicates that haploinsufficiency of ARID1B causes intellectual disability (ID) and autism spectrum disorder (ASD), but the neural function of ARID1B is largely unknown. Using both conditional and global *Arid1b* knockout mouse strains, we examined the role of ARID1B in neural progenitors. We detected an overall decrease in the proliferation of cortical and ventral neural progenitors following homozygous deletion of *Arid1b*, as well as altered cell cycle regulation and increased cell death. Each of these phenotypes was more pronounced in ventral neural progenitors. Furthermore, we observed decreased nuclear localization of β-catenin in *Arid1b*-deficient neurons. Conditional homozygous deletion of *Arid1b* in ventral neural progenitors led to pronounced ID- and ASD-like behaviors in mice, whereas the deletion in cortical neural progenitors resulted in minor cognitive deficits. This study suggests an essential role for ARID1B in forebrain neurogenesis and clarifies its more pronounced role in inhibitory neural progenitors. Our findings also provide insights into the pathogenesis of ID and ASD.

## Introduction

Intellectual disability (ID) and autism spectrum disorder (ASD) affect between one and three percent of the global population^1,2^. These and other related neurodevelopmental disorders represent a significant emotional and financial burden for affected individuals and their families^1^. Unfortunately, treatment for these conditions remains limited because many of the key molecular factors and their associated pathogenic mechanisms are still poorly understood. ARID1B is a sequence-specific, DNA-binding subunit in mammalian SWI/SNF or Brg1-associated factors (BAF) chromatin-remodeling complexes^3–5^. Neurogenetic studies have shown that *ARID1B* haploinsufficiency causes ID and ASD^6–8^. Because of the clear genetic linkage, defining the function of ARID1B in brain development is a crucial step toward understanding the neurological and developmental mechanisms responsible for these pathogenic phenotypes.

During brain development, forebrain excitatory and inhibitory neurons are generated in distinct brain regions and migrate along separate pathways before converging in the cerebral cortex. Excitatory neurons are born in the ventricular zone (VZ) of the developing cerebral cortex and migrate radially into the cortical plate, usually along radial glial processes^9–13^. Most inhibitory interneurons (GABAergic neurons) originate from a population of neural progenitors within the medial ganglionic eminence (MGE) of the ventral telencephalon and migrate tangentially into the dorsal telencephalon^14–16^. Cortical and ventral neural progenitors both need to be tightly regulated to ensure proper brain development as they have distinct and complementary roles in the mature brain and are each under the control of different pathways^17,18^. The balanced and coordinated function of pyramidal neurons and interneurons regulates excitatory and inhibitory tones in the brain. An imbalance of neuronal excitation and inhibition (E/I imbalance) in the developing brain underlies the neurological dysfunctions observed in ASD and ID^17–20^. Importantly, the numbers of these excitatory and inhibitory neurons are determined by the proliferation of cortical and ventral neural progenitors, respectively, in the developing brain^21^. We previously reported a significant decrease in the total number of GABAergic interneurons in the cerebral cortex of *Arid1b* haploinsufficient mice, suggesting that E/I imbalance may play a role in the pathology of *ARID1B*-related neurodevelopment disorders.

In order to separately identify the neurobiological function of ARID1B in excitatory and inhibitory neurons and their progenitors, we used conditional knockout mouse models to explicitly delete the *Arid1b* gene in either cortical or ventral neural progenitors. In this study, we utilize an *Emx1-Cre* driver line to conditionally delete *Arid1b* in cortical neural progenitors and a *Dlx5/6-Cre* driver to knockout *Arid1b* in ventral neural progenitors^22–25^. We report impaired proliferation in the cortical neural progenitor population and, to a greater extent, in ventral neural progenitors. This may be due to altered cell cycle regulation, as we observe decreased cell cycle speed in ventral neural progenitors with homozygous *Arid1b* deletion and a decreased rate of cell cycle re-entry in both cortical and ventral neural progenitors. In both progenitor populations we also report an increased number of apoptotic cells. Homozygous deletion of *Arid1b* in ventral inhibitory progenitors leads to ID- and ASD-like behavioral phenotypes, similar to those seen in *Arid1b* haploinsufficient mice^26–28^. Knockout of *Arid1b* in cortical excitatory progenitors, in contrast, has little effect on the mouse behaviors we measured. Taken together, *Arid1b* conditional homozygous deletion has an outsize effect on ventral progenitor proliferation, which is intricately linked to animal behavior, whereas homozygous loss of *Arid1b* in cortical progenitors gives rise to comparatively moderate neural and behavioral phenotypes.

## Results

### Decreased cortical progenitor proliferation in *Emx-Arid1b* mice

Conditional deletion of *Arid1b* in cortical progenitors was accomplished by crossing mice heterozygous for *Emx1-Cre*, which drives the expression of Cre recombinase in progenitors that give rise to cortical excitatory neurons^23^, with *Arid1b*^*LoxP/LoxP*^ mice^26^. Using Western blotting, we confirmed conditional knockout of ARID1B in mutant samples (Supplementary Fig. 1A).

**Figure 1:**
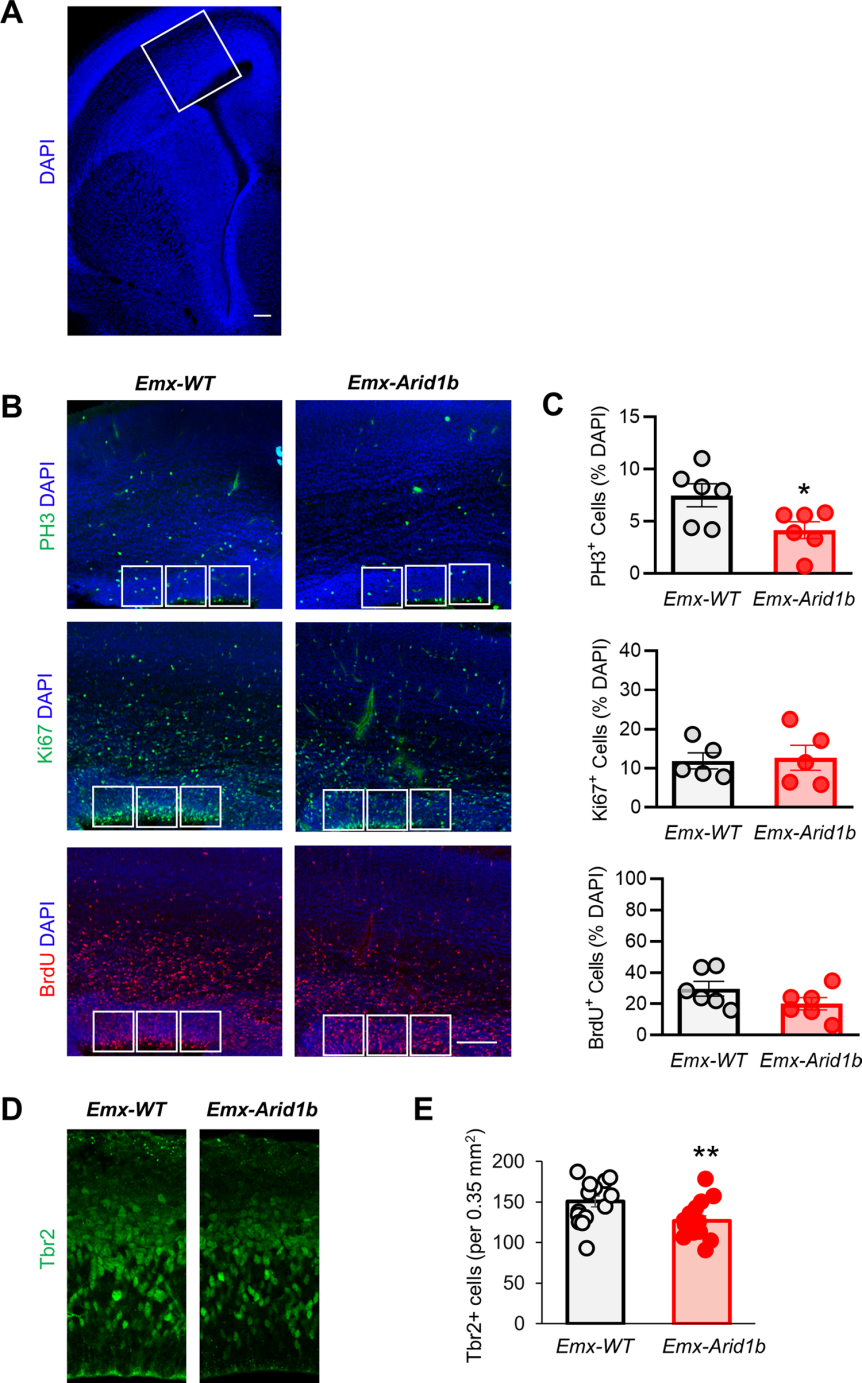
*Arid1b* deletion decreases cortical progenitor proliferation. (**A**) Representative low magnification image demonstrating the cortical brain sections examined in (**B**). (**B**) Immunostaining of coronal cerebral cortical sections from E14.5–15.5 control and *Arid1b*^*LoxP/LoxP*^*;Emx1-Cre* (*Emx-Arid1b*) brains with anti-phosphorylated Histone H3 (PH3), anti-Ki67 or anti-BrdU antibodies with DAPI co-stain. Representative images from both six control and *Emx-Arid1b* brains for PH3 and BrdU and five from each genotype for Ki67. Scale bars: 50 μm. White boxes (2.5 mm^2^) indicate regions of interest in the VZ of the developing cerebral cortex that were quantified and averaged for each animal. (**C**) Quantifications of the percentage of DAPI-positive cells co-labeled with the indicated antibody for panels in (**B**), respectively. N = 6 mice for each condition for PH3 and BrdU and N = 5 for each condition for Ki67. Sections were co-immunostained for DAPI, BrdU and PH3/Ki67. (**D**) Cortical sections of control and mutant samples were immunostained with an anti-Tbr2 antibody. (**E**) Quantification of (**D**). N = 15 mice for each condition. Statistical significance was determined by two-tailed Student’s *t* test. Error bars show standard error of the mean (SEM). *p < 0.05, **p < 0.01.

We first examined the proliferation of cortical neural progenitors in the VZ of the dorsal telencephalon from *Arid1b*^*LoxP/LoxP*^*;Emx1-Cre*^+^ (*Emx-Arid1b*) mice by immunostaining consecutive cortical sections from E14.5-E15.5 mouse embryos. Background cells were marked with a DAPI nuclear stain (Fig. 1A). DAPI labeling did not reveal any significant decrease in cells in the VZ of the developing cerebral cortex of *Emx-Arid1b* mice (263.8 cells), compared with *Arid1b*^*LoxP/LoxP*^*;Emx1-Cre*^−^ (*Emx-*WT) control littermates (274.0 cells) (Supplementary Fig. 2A). Staining with an anti-phosphorylated Histone-H3 (PH3) antibody, which is used to determine cells undergoing mitosis^29^, revealed a significant reduction (*p* < 0.05) in the percentage of DAPI-labeled mitotic cortical neural progenitors in *Emx-Arid1b* mice (4.162%), compared with controls (7.487%) (Fig. 1B,C). Staining for Ki67, which is present during all stages of the cell cycle and absent in quiescent (G_0_) cells^30,31^, showed no significant difference in the percentage of cells undergoing active proliferation in the VZ of *Emx-Arid1b* mice (12.67%), compared with controls (11.88%) (Fig. 1B,C). We also peritoneally injected all pregnant dams with bromodeoxyuridine (BrdU), a thymidine analog that is incorporated into dividing cells during DNA replication^32–34^, and found no significant decrease in the percentage of BrdU-positive cortical neural progenitors in *Emx-Arid1b* mice harvested 1 h post-injection (20.04%), compared with controls (29.67%) (Fig. 1B,C). To further examine the cortical progenitor population, we immunostained sections of the developing cerebral cortex with an antibody against a marker for intermediate progenitors, Tbr2, and discovered a significant decrease in this population in *Emx-Arid1b* mice (126.9 cells), compared with controls (150.4 cells) (Fig. 1D,E).

**Figure 2:**
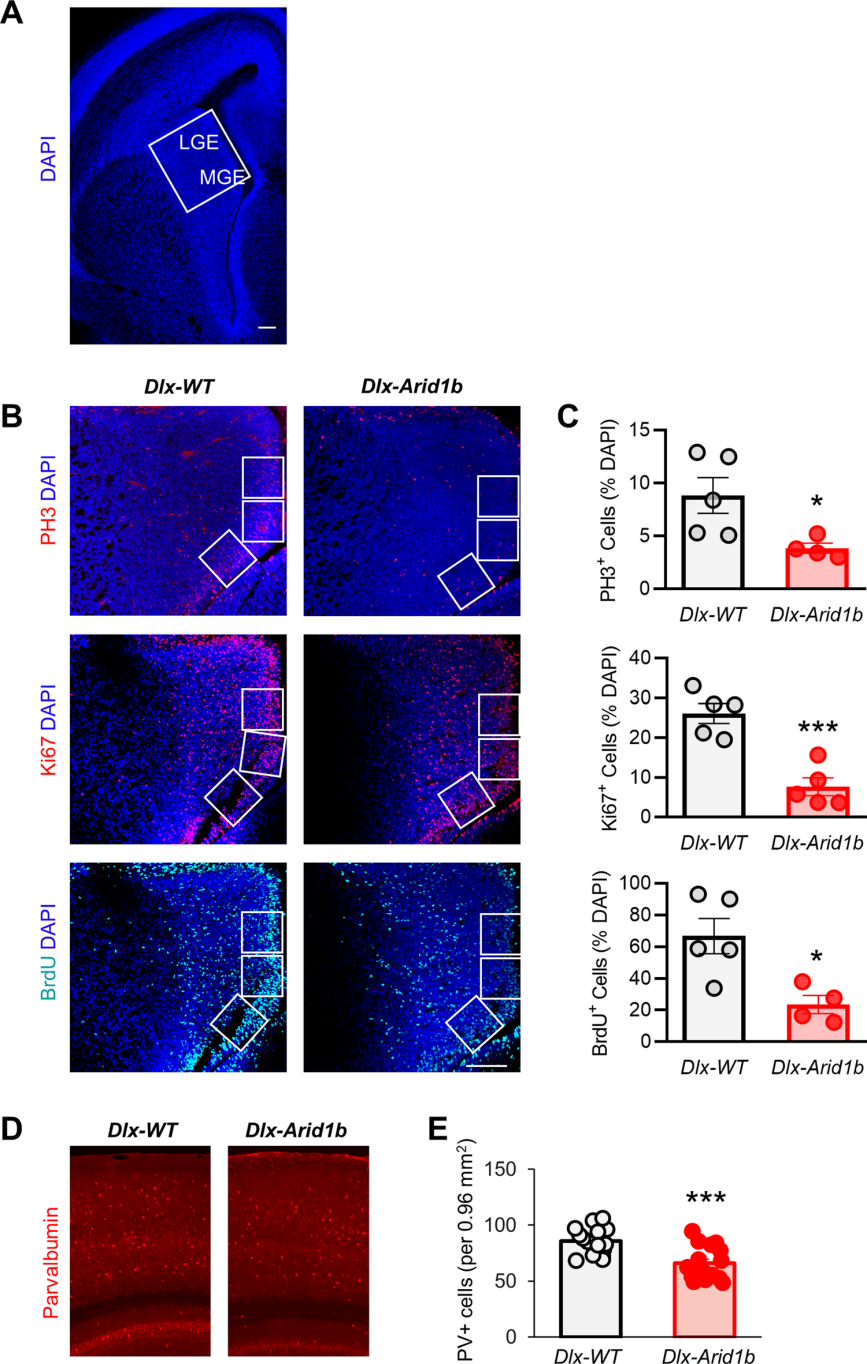
*Arid1b* deletion greatly decreases ventral progenitor proliferation. (**A**) Representative low magnification image demonstrating the location of brain sections examined in (**B**), including labeling of the LGE and MGE. (**B**) Immunostaining of coronal sections from E14.5 to 15.5 control and *Arid1b*^*LoxP/LoxP*^*;Dlx5/6-Cre* (*Dlx-Arid1b*) brains with anti-phosphorylated Histone H3 (PH3), anti-Ki67 or anti-BrdU antibodies with DAPI co-stain. Representative images from both six control and *Dlx-Arid1b* brains for PH3, five from each genotype for Ki67, and five control and four *Dlx-Arid1b* brains for BrdU. Scale bars: 50 μm. White boxes (2.5 mm^2^) indicate regions of interest in the VZ of the MGE that were quantified and averaged for each animal. (**C**) Quantifications of the percentage of DAPI-positive cells co-labeled with the indicated antibody for panels in (**B**). N = 6 mice for each condition for PH3, N = 5 for each genotype in Ki67, and N = 5 mice for control and 4 for BrdU. Sections were co-immunostained for DAPI, BrdU and PH3/Ki67. (**D**) Cortical sections of P30 *Dlx-Arid1b* and their WT controls were immunostained with a parvalbumin antibody. (**E**) Quantification of (**D**). N = 15 mice for each condition. Statistical significance was determined by two-tailed Student’s *t* test. Error bars show standard error of the mean (SEM). *p < 0.05, ***p < 0.001.

### Impaired ventral progenitor proliferation in *Dlx-Arid1b* mice

We utilized *Arid1b*^*LoxP/LoxP*^*;Dlx5/6-Cre*^+^ (*Dlx-Arid1b*) mice to examine the effects of homozygous deletion of *Arid1b* in ventral progenitors that give rise to cortical GABAergic inhibitory interneurons. Conditional knockout was confirmed by Western blotting (Supplementary Fig. 1B). In E14.5-E15.5 *Dlx-Arid1b* mice, we first showed that *Dlx-Arid1b* mice do not display a reduction in the number of likely progenitor cells in the VZ of the MGE (344.3 cells), compared with *Arid1b*^*LoxP/LoxP*^*;Dlx5/6-Cre*^−^ (*Dlx-*WT) control littermates (330.7 cells) (Supplementary Fig. 2B). We next examined ventral neural progenitor proliferation by immunostaining for PH3, Ki67 and BrdU in the VZ of the MGE. Background cells were marked by DAPI (Fig. 2A). We observed a significant decrease (*p* < 0.05) in the percentage of cells positive for PH3 (3.852%) (Fig. 2B,C), as well as a significant decrease (*p* < 0.001) in the percentage of cells expressing Ki67 (7.643%) (Fig. 2B,C), both compared with controls (8.829% and 26.090%, respectively). These indicate large reductions in the proportion of mitotic and actively proliferating ventral neural progenitors in these mice. Following BrdU injection into all *Arid1b*^*LoxP/LoxP*^*;Dlx5/6-Cre*^+^ (*Dlx-Arid1b*) pregnant dams, displayed a significant decrease (*p* < 0.05) in the percentage of cells positive for BrdU in the VZ of the MGE (23.57%), compared with controls (66.76%) (Fig. 2B,C). Consequently, we also observed a significant decrease (*p* < 0.001) in parvalbumin (PV)-positive interneurons in the cerebral cortex of P30 *Dlx-Arid1b* mice, compared with controls (Fig. 2D,E). We did not, however, detect any change in the total number of neurons in the cerebral cortex at P30, as shown by NeuN immunostaining (Supplemental Fig. 3A,B). Together, these results show a reduction in the number of proliferating ventral neural progenitors in the MGE, leading to a lower number of PV-positive interneurons in the adult cerebral cortex.

**Figure 3:**
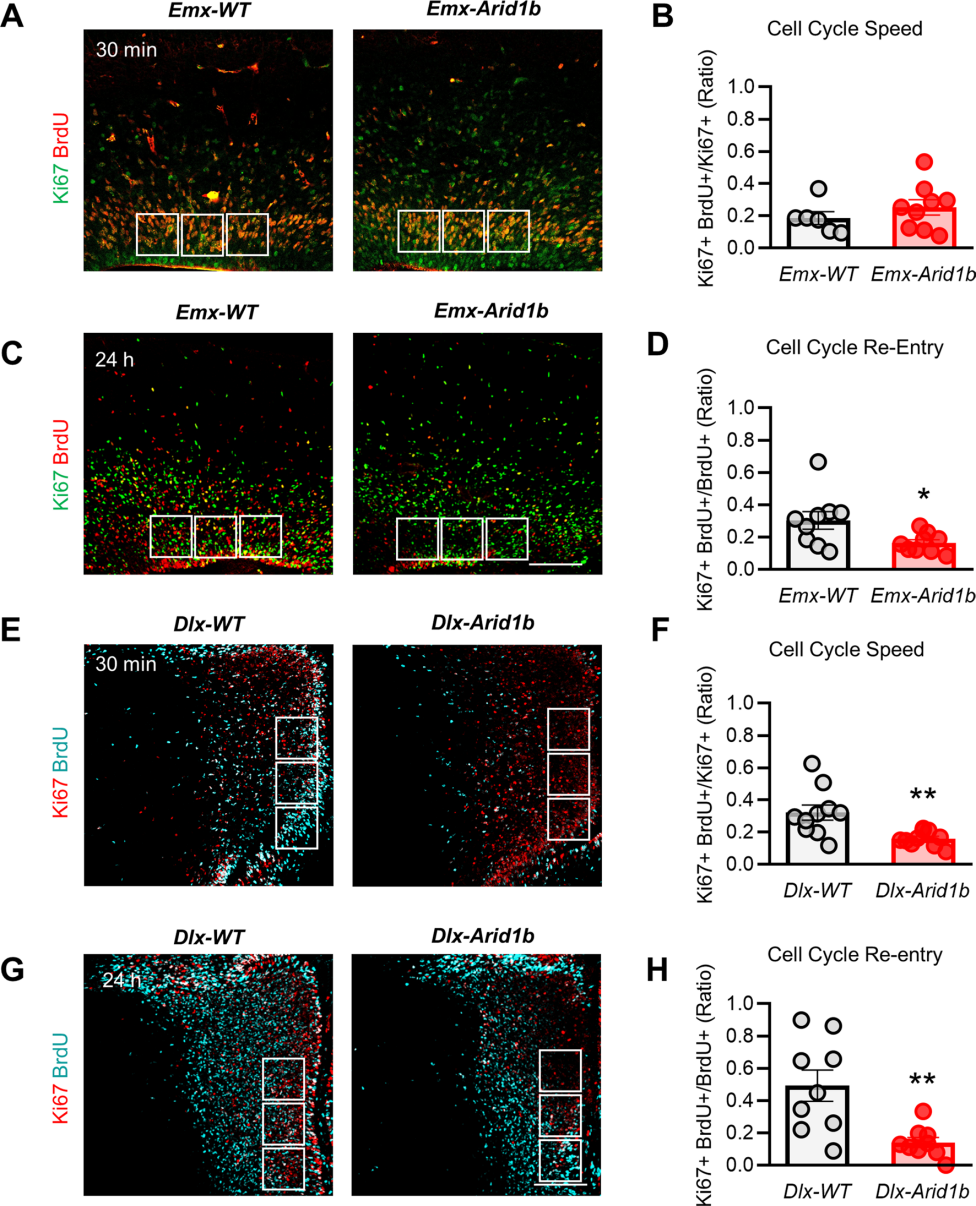
ARID1B regulates the cell cycle in neural progenitors. (**A**) and (**E**) E14–16 control and *Emx-Arid1b* or *Dlx-Arid1b* mice were pulse-labeled with BrdU for 30 min, and then brain regions containing the cerebral cortex were collected and immunostained using BrdU and Ki67 antibodies. Scale bar: 50 μm. White boxes (2.5 mm^2^) indicate regions of interest in the VZ of the developing cerebral cortex (for *Emx-Arid1b* mice) or MGE (for *Dlx-Arid1b* mice) that were quantified and averaged for each animal. (**B**) and (**F**) Quantification of cell cycle speed from (**A**) and (**E**). The cell cycle speed was defined as the fraction of BrdU- and Ki67-double-positive cells in the total Ki67-positive pool in the cerebral cortex. N = 6 control and 9 *Emx-Arid1b* mice for (**B**) and N = 10 for each genotype for (**F**). Statistical significance was determined by two-tailed Student’s *t* test. Error bars show SEM. **p < 0.01. (**C**) and (**G**) E14-16 control and *Emx-Arid1b* or *Dlx-Arid1b* mice were pulse-labeled with BrdU for 24 h and then brains were collected for immunostaining with BrdU and Ki67 antibodies. Scale bar: 50 μm. (**D**) and (**H**) The index of cell cycle re-entry was calculated as the fraction of BrdU- and Ki67-double-positive cells in total BrdU-positive pool. N = 9 mice for each genotype from both (**D**) and (**H**). Statistical significance was determined by two-tailed Student’s *t* test. Error bars show SEM. *p < 0.05, **p < 0.01.

### ARID1B regulates cell cycle progression in both cortical and ventral neural progenitors

Next, we explored the effects of conditional *Arid1b* knockout on cell cycle progression. Cell cycle speed and cell cycle re-entry were examined in both *Emx-Arid1b* and *Dlx-Arid1b* mice. Cell cycle speed was assessed by first injecting pregnant dams with BrdU 30 min prior to removal of E14.5 embryos, followed by immunostaining of brain sections using both anti-BrdU and anti-Ki67 antibodies. The ratio of Ki67-BrdU-double-positive cells to the total number of Ki67-positive cells, essentially the proportion of actively proliferating cells that entered S-phase in a 30 min time span, provides a quantifiable estimate of cell cycle speed^35,36^. Homozygous knockout of *Arid1b* in cortical neural progenitors had no significant impact on cell cycle speed in the VZ of the developing cerebral cortex (*Emx-Arid1b*: 0.2526 vs. *Emx*-WT: 0.1867) (Fig. 3A,B). In the ventral neural progenitor population, however, *Arid1b* deletion led to a 50.33% reduction (*p* < 0.01) in this measurement of cell cycle speed, compared with control littermates (*Dlx-Arid1b*: 0.1599 vs. *Dlx*-WT: 0.3213) (Fig. 3E,F).

**Figure 4:**
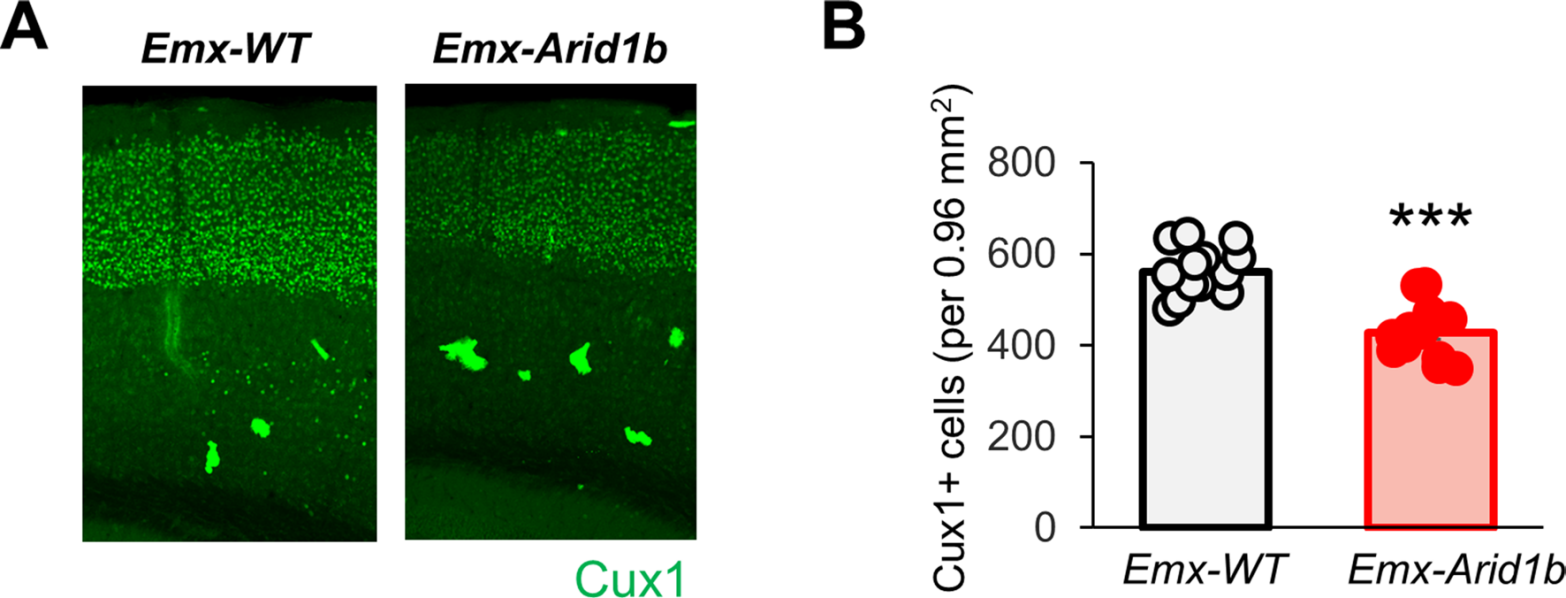
*Arid1b* deletion in cortical progenitors leads to a reduction in upper layer neurons. (**A**) Cortical sections of P30 control and *Emx-Arid1b* mice were immunostained using an anti-Cux1 antibody. (**B**) Quantification of (**A**). The number of Cux1-positive neurons was decreased in *Emx-Arid1b* mice. N = 15 for each genotype. Statistical significance was determined by two-tailed Student’s *t* test. Error bars show SEM. ***p < 0.001.

To quantify cell cycle re-entry, we injected pregnant dams with BrdU 24 h prior to euthanasia and then co-immunostained E15.5 embryonic brains with anti-Ki67 and anti-BrdU antibodies. The ratio of Ki67-BrdU-double-positive cells to the total number of BrdU-positive cells provides a measure of the cells that re-enter the cell cycle^35,36^. There was a 46.06% (*p* < 0.05) reduction in BrdU-positive cortical neural progenitors that express measurable levels of Ki67 in *Emx-Arid1b* mice (0.1637), compared with controls (0.3029), which indicates impaired cell cycle re-entry (Fig. 3C,D). We further examined the effects of this reduction in cell cycle re-entry amongst *Emx-Arid1b* mice by immunostaining the cerebral cortex for a marker of superficial pyramidal neurons, Cux1, in P30 animals, and found that there is a significant decrease (*p* < 0.001) in Cux1-positive neurons in *Emx-Arid1b* mice (427.1), compared with controls (559.7) (Fig. 4A,B). Ventral neural progenitors also showed an impairment in cell cycle re-entry, as they had fewer BrdU-positive ventral neural progenitors positive to Ki67 (− 71.48%) in *Dlx-Arid1b* mice (0.1405), compared with *Dlx*-WT littermates (0.4925) (Fig. 3G,H).

### Conditional homozygous deletion of *Arid1b* leads to increased apoptosis in cortical and ventral neural progenitors

**Figure 5:**
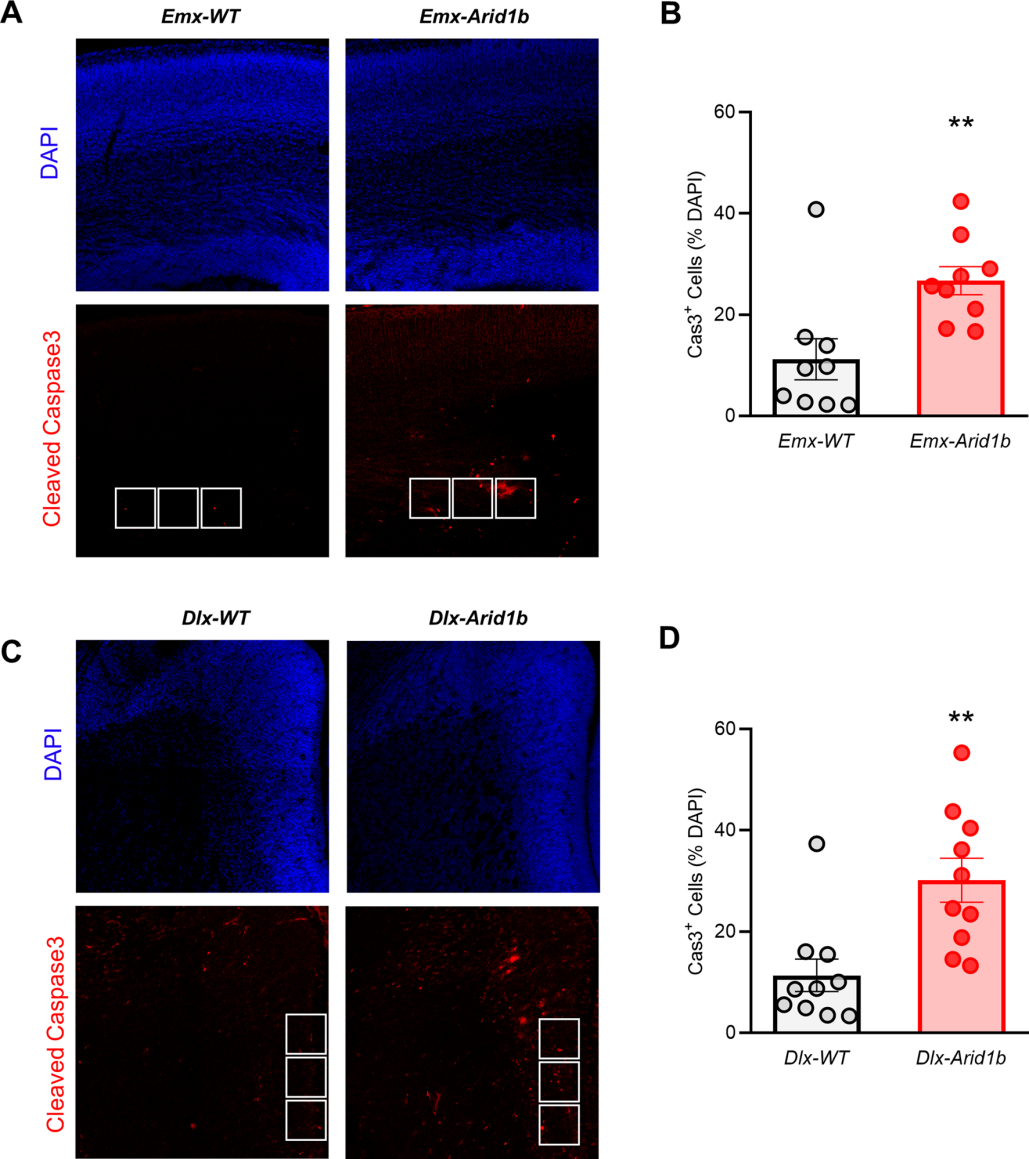
*Arid1b* deletion increases apoptosis in cortical and ventral neural progenitor pools. (**A**) and (**C**) Cell death was assessed in E14-16 control and *Emx-Arid1b* or *Dlx-Arid1b* mutant neural progenitors by immunostaining with a cleaved caspase-3 (cl.-Cas 3) antibody. Representative images from both nine control and *Emx-Arid1b* brains for (**A**) and ten from each genotype for (**C**). White boxes (2.5 mm^2^) indicate regions of interest in the VZ of the developing cerebral cortex (for *Emx-Arid1b* mice) or MGE (for *Dlx-Arid1b* mice) that were quantified and averaged for each animal. (**B**) and (**D**) Quantification of apoptotic cells (the percentage of DAPI cells positive for cl.-Cas 3) from (**A**) and (**C**), respectively. N = 9 mice for each genotype for (**A**) and N = 10 mice for each genotype for (**C**). Statistical significance was determined by two-tailed Student’s *t* test. Error bars show SEM. **p < 0.01.

In addition to examining the proliferation of cortical and ventral neural progenitors, we assessed apoptotic progenitors in the E14.5-E15.5 developing cerebral cortex and ventral telencephalon in conditional *Arid1b* mutants. We used an anti-cleaved-Caspase 3 (anti-cl.-Cas 3) antibody to determine the relative percentage of cells undergoing apoptosis. In *Emx-Arid1b* mice, the percentage of DAPI-labeled cells in the VZ of the dorsal telencephalon co-expressing cl.-Cas 3 (26.72%) was significantly higher (*p* < 0.01) than in wild type mice (11.20%) (Fig. 5A,B). In *Dlx-Arid1b* mice, the percentage of cells expressing cl.-Cas 3 in the VZ of the MGE (30.13%) was likewise significantly higher than in controls (11.36%) (Fig. 5C,D). From these results we conclude that homozygous deletion of *Arid1b* leads to a comparable increase in apoptosis among both cortical and ventral neural progenitor populations.

### *Arid1b* knockout decreases β-catenin nuclear localization in vitro and in vivo

**Figure 6:**
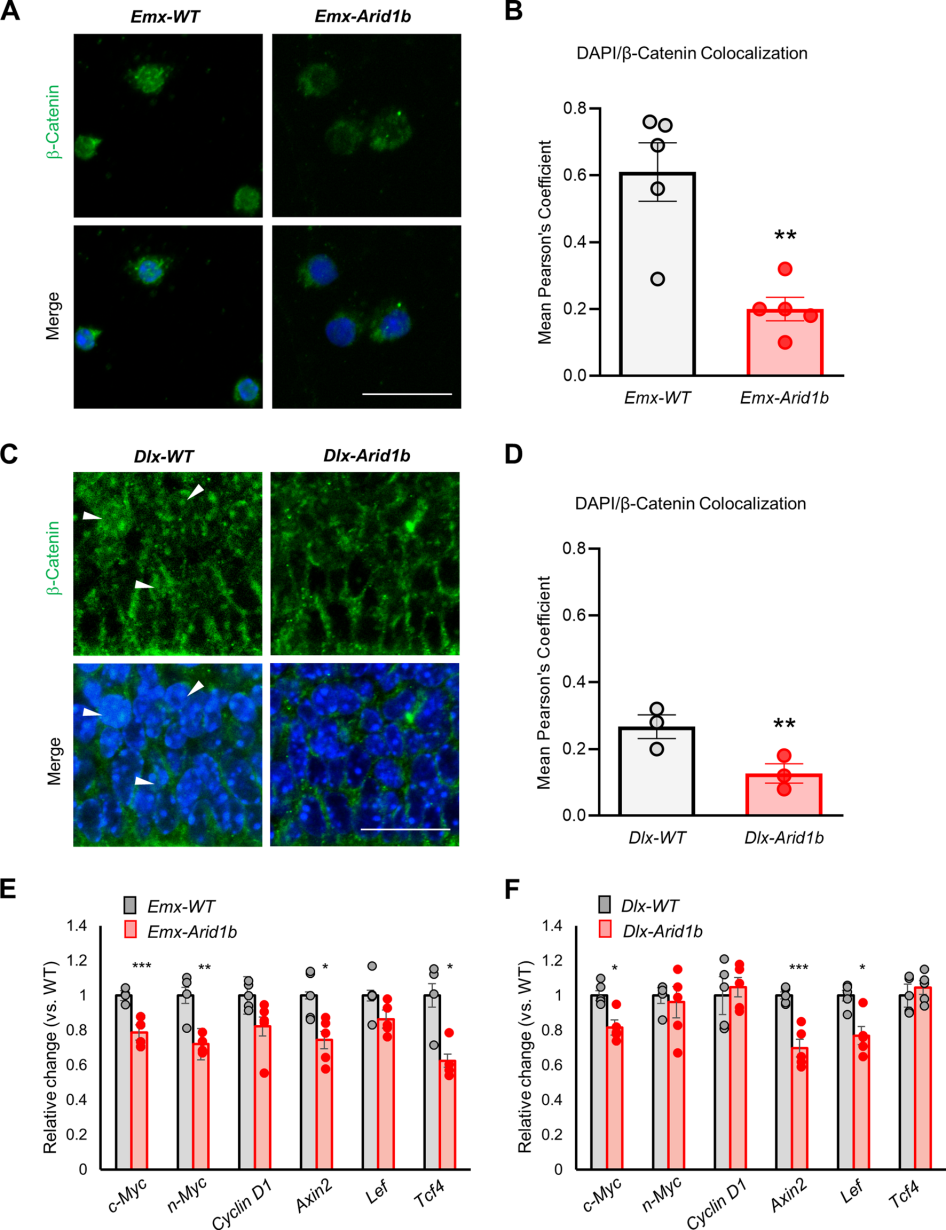
Knockout of *Arid1b* reduces β-catenin nuclear localization. (**A**) and (**C**) Nuclear localization of β-catenin was assessed in DIV6 control and *Arid1b*^−/−^ mouse primary neuronal cultures derived from E12.5 to E14.5 embryonic MGEs by immunostaining with DAPI and an antibody against β-catenin. Representative images from N = 5 mice from each genotype for (**A**) and 3 mice from each genotype for (**C**). Scale bar: 50 μm. White arrows indicate nuclei qualitatively positive for β-catenin. (**B**) and (**D**) Quantification of (**A**) and (**C**), respectively. Mean corrected Pearson’s coefficient from object-corrected fluorescence colocalization of DAPI and anti-β-cat is plotted. N = 5 mice from each genotype for (**B**) and 3 mice from each genotype for (**D**). (**E**) and (**F**) Expression of Wnt/β-catenin signaling components was assessed by RT-PCR in E12.5–E14.5 *Emx-Arid1b* and *Dlx-Arid1b* brains. mRNA levels were quantified from 5 mice for each genotype. Statistical significance was determined by two-tailed Student’s *t* test. Error bars show SEM. *p < 0.05, **p < 0.01, ***p < 0.001.

We previously showed that *Arid1b* haploinsufficiency leads to a significant reduction in β-catenin protein expression and the expression of several Wnt/β-catenin signaling target genes in the ventral telencephalon^26^. Two other groups have also shown that ARID1B is involved in the regulation of β-catenin signaling^37,38^. Here we assessed the localization pattern of the transcription factor β-catenin because it requires nuclear translocation in order to regulate downstream gene expression. We cultured primary neural progenitors from the MGE of E12.5 *Arid1b*^+*/*+^ and *Arid1b*^−/−^ brains, and immunostained unstimulated cells using an anti-β-catenin antibody. Qualitative analysis revealed decreased β-catenin in the DAPI-stained nuclei of *Arid1b*^−/−^ cells, compared with *Arid1b*^+*/*+^ controls. We determined the object-corrected fluorescence colocalization of β-catenin and DAPI^39^. The mean Pearson’s coefficient for *Arid1b*^+*/*+^ cultures was 0.61 and 0.20 for *Arid1b*^−/−^ cultures, indicating significantly reduced nuclear β-catenin in the absence of ARID1B (*p* < 0.01) (Fig. 6A,B). Next, we examined nuclear β-catenin localization in the mouse brain by immunostaining MGEs of E12.5 *Dlx-Arid1b* mice and comparing DAPI-β-catenin colocalization with control MGEs. Similar to what we observed in vitro, we detected significantly less (*p* < 0.01) overlapping β-catenin and DAPI staining in *Dlx-Arid1b* MGEs than in controls (Fig. 6C,D). Pearson’s coefficient of colocalization in *Dlx-Arid1b* MGEs was lower (0.03) than that in controls (0.27). Furthermore, we examined the expression of β-catenin downstream genes including *c-Myc, n-Myc, Cyclin D1, Axin2, Lef, and Tcf4*. The levels of *c-Myc, n-Myc, Axin2,* and *Tcf4* were deceased in *Emx-Arid1b* samples compared with controls (Fig. 6E). Similarly, the *c-Myc, Axin2*, and *Lef* levels were reduced in *Dlx-Arid1b* mutants (Fig. 6F). Taken together, we conclude that loss of *Arid1b* leads to aberrant β-catenin signaling in the brain.

### Cortical progenitor-specific deletion of *Arid1b* in neural behavior

**Figure 7:**
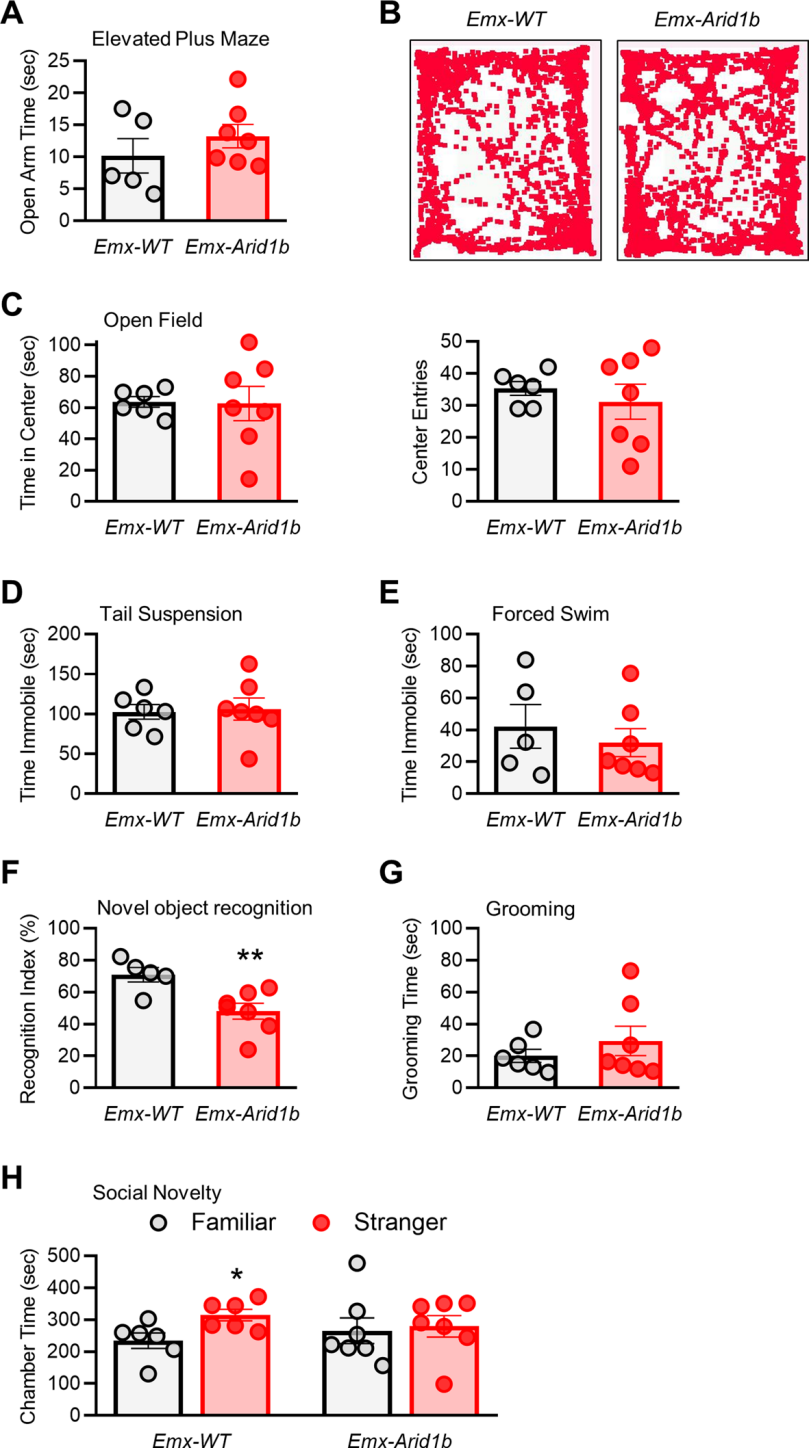
Effects of conditional deletion of *Arid1b* in cortical neural progenitors on behavior. (**A**) In the elevated plus maze, *Emx-Arid1b* mice spend the same amount of time in the open arm as controls. N = 6 mice for control and N = 7 mice for *Emx-Arid1b*. Statistical significance was determined by two-tailed Student’s *t* test. Error bars show SEM. (**B**) No significant difference in immobility times in the tail suspension test between controls and *Emx-Arid1b* mice. N = 6 mice for control and N = 7 mice for *Emx-Arid1b*. Statistical significance was determined by two-tailed Student’s *t* test. Error bars show SEM. (**C**) In the forced swim test there was no significant difference in immobility time between control and *Emx-Arid1b*. N = 6 mice for control and N = 7 mice for *Emx-Arid1b*. Statistical significance was determined by two-tailed Student’s *t* test. Error bars show SEM. (**D**) Representative traces from (**E**). (**E**) No significant difference in the total time spent in the center or the number of entries into the center in the open field test. N = 6 mice for control and N = 7 mice for *Emx-Arid1b*. Statistical significance was determined by two-tailed Student’s *t* test. Error bars show SEM. (**F**) No significant change in grooming time in a 10-min session between control and *Emx-Arid1b* mice. N = 6 mice for control and N = 7 mice for *Emx-Arid1b*. Statistical significance was determined by two-tailed Student’s *t* test. Error bars show SEM. (**G**) *Emx-Arid1b* mice demonstrate impaired novel object recognition. Recognition index indicates the percentage of time the test mouse interacted with a novel object compared to a familiar one. N = 5 mice for control and N = 7 mice for *Emx-Arid1b*. Statistical significance was determined by two-tailed Student’s *t* test. Error bars show SEM. (**H**) Social behavior in *Emx-Arid1b* mice was tested by the three-chamber social assay. N = 6 mice for control and N = 7 mice for *Emx-Arid1b*. Statistical significance was determined by two-tailed Student’s *t* test. Error bars show SEM. *p < 0.05, **p < 0.01.

We next examined the behavioral outcomes of homozygous deletion of *Arid1b* in excitatory and inhibitory neurons and their progenitors in 8–10-week-old mice. Anxiety is a frequent comorbid condition with ASD in humans^40–42^, therefore, we first examined anxiety-like behaviors in *Emx-Arid1b* mice by performing the elevated plus maze and open field assays. *Emx-Arid1b* mice spent the same amount of time in the open arms of the elevated plus maze (13.24 s) as controls (10.16 s) (Fig. 7A). In the open field test, *Emx-Arid1b* mice entered, and spent the same amount of time in, the center portion of the open field apparatus (62.52 s), compared with wild type littermates (63.69 s) (Fig. 7B,C). From these results we concluded that *Emx-Arid1b* mice display no appreciable anxiety-like behaviors. We next assessed depression-like behaviors using the tail suspension and forced swim tests. *Emx-Arid1b* mice did not spend a discernably different amount of time immobile in either of these assays, compared with control mice (Fig. 7D,E). These results show that homozygous deletion of *Arid1b* in cortical neural progenitors does not appear to engender emotional symptoms such as anxiety-like and depression-like behaviors in mice.

We also investigated the effects of homozygous *Arid1b* deletion in cortical neural progenitors on cognitive performance in the novel object recognition task. *Arid1b*^+*/*+^*;Emx1-Cre* (*Emx*-WT) littermates displayed a recognition index of 70.88%, indicative of a stronger preference for interaction with the novel object (Fig. 7F). *Emx-Arid1b* mice, on the other hand, exhibited a recognition index of just 48.05% in this task, which indicates no real preference for a novel object versus a familiar one in an otherwise empty open field. Thus, complete *Arid1b* deletion in cortical progenitors leads to cognitive impairments in mice. Additionally, the measurement of grooming time showed no significant difference between *Emx-Arid1b* (29.40 s) and *Emx*-WT mice (19.98 s), suggesting that stereotypic behavior is not associated with *Arid1b* deletion in cortical progenitors (Fig. 7G). Furthermore, *Emx-Arid1b* demonstrated social novelty deficits compared with their *Emx*-WT littermates (Fig. 7H), which may further reflect the cognitive impairments observed in the novel object recognition test, or provide further evidence of an ASD-like social phenotype.

### Conditional knockout of *Arid1b* in ventral neural progenitors results in emotional and social impairments

**Figure 8:**
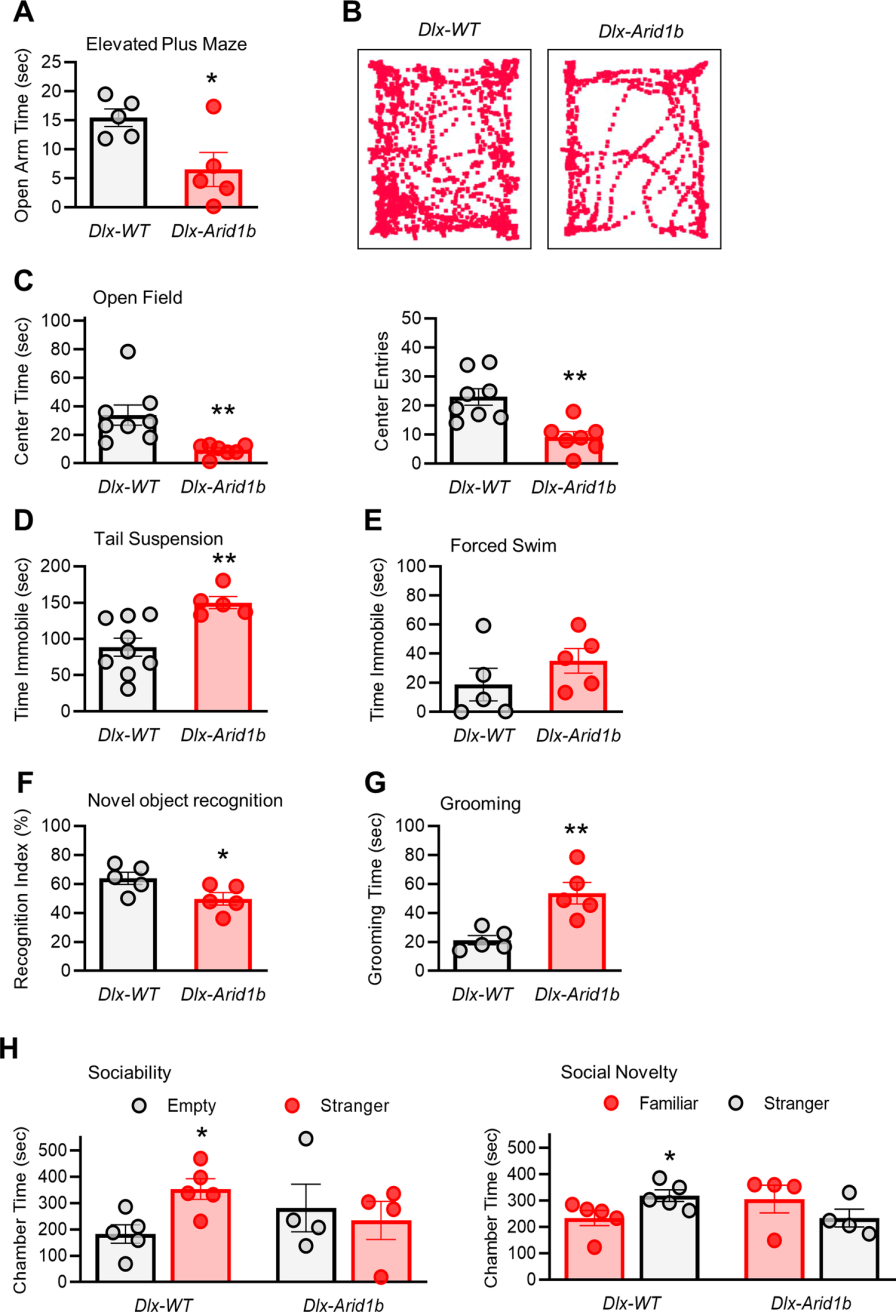
Effects of conditional deletion of *Arid1b* in ventral neural progenitors on behavior. (**A**) In the elevated plus maze, *Dlx-Arid1b* mice spend significantly more time in the open arms than controls. N = 5 mice from each genotype. Statistical significance was determined by two-tailed Student’s *t* test. Error bars show SEM. (**B**) Representative traces from (**C**). (**C**) *Dlx-Arid1b* mice spend significantly less total time in the center and enter the center fewer times in the open field test. N = 10 mice for control and N = 7 mice for *Dlx-Arid1b*. Statistical significance was determined by two-tailed Student’s *t* test. Error bars show SEM. (**D**) *Dlx-Arid1b* spend significantly more time immobile in the tail suspension test than controls. N = 9 mice for control and N = 5 mice for *Dlx-Arid1b*. Statistical significance was determined by two-tailed Student’s *t* test. Error bars show SEM. (**E**) In the forced swim test there was no significant difference in the immobility time of control and *Dlx-Arid1b* mice. N = 5 mice for each genotype. Statistical significance was determined by two-tailed Student’s *t* test. Error bars show SEM. (**F**) Significantly lower recognition index in the novel object recognition test in *Dlx-Arid1b* compared with control mice. N = 5 mice for each genotype. Statistical significance was determined by two-tailed Student’s *t* test. Error bars show SEM. (**G**) *Dlx-Arid1b* mice spend significantly more time grooming than controls in a 10-min session. N = 5 mice for each genotype. Statistical significance was determined by two-tailed Student’s *t* test. Error bars show SEM. (**H**) *Dlx-Arid1b* mice demonstrate significantly impaired sociability and social novelty behaviors compared with controls. N = 5 mice for control and N = 4 mice for *Dlx-Arid1b*. Statistical significance was determined by two-tailed Student’s *t* test. Error bars show SEM. *p < 0.05, **p < 0.01.

We also examined ASD- and ID-related behaviors in mice in which *Arid1b* is deleted in ventral neural progenitors. In the elevated plus maze test, *Dlx-Arid1b* mice spent significantly less time in the open arm (6.529 s) than their wild type littermates (15.440 s) (Fig. 8A). In the open field assay, *Dlx-Arid1b* mice entered and spent significantly less time in the center area of the apparatus (9.347 s), compared to controls (33.88 s) (Fig. 8B,C), which is indicative of anxiety-like behavior. *Dlx-Arid1b* mice also spent significantly more time immobile in the tail suspension assay (150.10 s), compared with control littermates (88.49 s) (Fig. 8D). The forced swim test also showed a similar trend of immobility in *Dlx-Arid1b* mice, but did not reach statistical significance (Fig. 8E). These results suggest that deletion of *Arid1b* in ventral neural progenitors may be sufficient to cause anxiety-like and depression-like behaviors in mice. In the novel object recognition test, *Dlx-Arid1b* mice also exhibited a significant difference in recognition index (49.75%), compared with controls (63.87%) (Fig. 8F). This suggests that, like *Emx-Arid1b* mice, *Dlx-Arid1b* mice exhibit an appreciable deficit in recognition memory, though, ostensibly, not to the same degree.

*Dlx-Arid1b* mice exhibit a strong tendency toward repetitive behaviors, as evidenced by them spending more than 2.5 times as much time grooming themselves during a ten-minute window (53.61 s) than their wild type littermates (21.23 s) (Fig. 8G). In the sociability test using the three-chamber paradigm, control mice spent more time in the chamber containing a stranger mouse than in the empty chamber (*p* < 0.05). *Dlx-Arid1b* mice, however, showed no preference for either the empty or stranger chamber (Fig. 8H). In the social novelty test, *Dlx-Arid1b* mice did not display a significant difference in their time spent between chambers containing familiar and stranger mice, while control mice exhibited a clear preference for stranger (*p* < 0.05). Together with the grooming test results, these data show that homozygous deletion of *Arid1b* in the ventral neural progenitors contributes to social and stereotypic behaviors, key hallmarks of ASD-like behavior in mice.

## Discussion

As we and other groups have recently reported, deletion of one *Arid1b* allele in mice is sufficient to cause significant behavioral deficiencies, recapitulating ID and ASD behavioral phenotypes^26–28^. These mouse models of *Arid1b* haploinsufficiency are useful because they mirror the gene dosage effects seen in human patients, but are less useful for deciphering the neurobiological function of the ARID1B protein, whose levels remain above fifty percent in *Arid1b* mutant heterozygotes, compared to their wild-type littermates^26–28^. Our group showed that adult heterozygous *Arid1b* knockout mice display a significant reduction in the overall number of their cortical interneurons, with no major changes in cortical pyramidal neurons^26^. It is unclear, however, what causes the decrease in cortical inhibitory neurons in the *Arid1b* haploinsufficient condition. We surmised that this is most likely due to defective proliferation and/or impaired cell survival in the ventral neural progenitor population.

Several BAF subunits have been shown to influence cell cycle progression and cell division^43–48^. In this study, we show that conditional homozygous knockout of *Arid1b* leads to an overall decrease in the number of actively proliferating and/or mitotic cells, as well as fewer newborn neurons or neural progenitors in the MGE. We also report that loss of *Arid1b* in ventral neural progenitors leads to increased apoptosis in the MGE. The defects in proliferation are due, at least in part, to improper cell cycle regulation, as we observe reduced cell cycle speed and a lower rate of cell cycle re-entry in *Arid1b*-deleted ventral neural progenitors. Somewhat unexpectedly, homozygous deletion of *Arid1b* in cortical neural progenitors that produce cortical pyramidal neurons also leads to significant defects in cell proliferation and survival, although these impairments are not as severe as what we observe with *Arid1b* deletion in the ventral telencephalon. It appears that *Arid1b* deletion in cortical progenitors leads to pre-mitotic cell cycle arrest, as the number of progenitors apparently active in the cell cycle is not altered. These findings suggest that *Arid1b* plays a more substantial role in regulating proliferation of ventral neural progenitors than it does in cortical neural progenitors. Homozygous deletion of *Arid1b* leads to similar increases in apoptosis in both cortical and ventral neural progenitor populations. Thus, the role of ARID1B in cell survival is likely similar in both progenitor subgroups and potentially elsewhere in the brain or periphery. In the current study we examine the effects of ARID1B during a relatively brief developmental window (E14.5–E15.5), but during this time frame many changes take place. Although we did not observe any striking differences in the metrics we used to evaluate proliferation and survival between E14.5 and E15.5 brains, these are distinct developmental phases, and a more careful examination of these timepoints, individually, may be warranted. Overall, it remains to be determined whether ARID1B’s functions in regulating cell proliferation or survival is likely to play a larger role in pathology of *ARID1B*-related neurodevelopmental disorders.

We show that homozygous deletion of *Arid1b* leads to reduced nuclear localization of β-catenin in ventral neural progenitors. Brahma-related gene-1 (BRG1), encoded by *SMARCA4*, is the core subunit of the mammalian BAF chromatin remodeling complex^49,50^ and interacts directly with ARID1B in the nucleus^51,52^. BRG1 has also been shown to physically interact with nuclear β-catenin at target gene promoters and facilitate transcriptional activation, most likely via chromatin remodeling^53^. A recent report confirms that ARID1B regulates Wnt/β-catenin signaling in HEK293T cells in a BRG1-dependent manner^54^. Vasileiou et al. also reported that expression of β-catenin target genes is increased in peripheral blood lymphocytes taken from human patients with *ARID1B* loss-of-function mutations^37^. Furthermore, ARID1B interacts with β-catenin in a human osteosarcoma cell line, and the ARID1B-β-catenin interaction in these cells is mediated by BRG1^37^. This suggests a negative role for ARID1B and BRG1 in regulating β-catenin activity. Yet another group’s findings support this conclusion, as they demonstrated that shRNA-mediated knockdown of *Arid1b* leads to increased expression of β-catenin target genes in *Stat3* knockout, sciatic-nerve-derived neurofibroma spheres^38^. One further group did report a contradictory finding, however, showing that BRG1 enhances β-catenin-mediated gene expression by binding to β-catenin at target gene promoters and facilitating chromatin remodeling^53^. Our recent study involving global heterozygous knockout of *Arid1b* agrees more with this finding, as we see significant decreases in the mRNA expression levels of several β-catenin target genes in the ventral telencephalon^26^. Multiple studies have shown broad diversity in the configuration of BAF complexes, depending on cell types^43,46,48,55–61^. Different switches in subunit composition play a large role in determining which genes are targeted by BAF complexes and how they mediate transcription. For this reason, the effects of *Arid1b* knockdown in non-neural progenitor or non-neuronal populations may be entirely different than what is observed in the brain. It is entirely possible that ARID1B acts to repress β-catenin in a BRG1-dependent manner in peripheral blood lymphocytes and tumor cell lines and enhance β-catenin transcriptional activation in the developing brain. This is important because proper control of β-catenin signaling is necessary for the proliferation of neural progenitors in the ventral telencephalon^62^. Therefore, the reduction in ventral progenitor proliferation in *Dlx-Arid1b* mice may be, at least in part, due to dysregulation of β-catenin signaling.

In addition to regulating Wnt/β-catenin signaling, we previously reported that *Arid1b* haploinsufficiency leads to disruptions in the epigenetic regulation of the *Pvalb* gene, which encodes PV^26^. This may explain the reduction in cortical PV-positive interneurons we observed here in *Dlx-Arid1b* mice, and in the aforementioned study when we performed heterozygous deletion of *Arid1b* in ventral progenitors^26^. As a member of the BAF chromatin remodeling complex, it should come as no surprise that deletion of *Arid1b* would have a far-reaching impact on the expression of many genes. Indeed, two groups performed RNA-sequencing on brains taken from *Arid1b* haploinsufficient mice and reported differential regulation of a large number of genes, including many ASD-related genes^27,28^. Therefore, the underlying causes of ARID1B-related behavioral phenotypes are likely varied and overlapping.

*Arid1b* haploinsufficient mice demonstrate marked ID- and ASD-like characteristics^26–28^, making them a useful mouse model for studying neurodevelopmental disorders. In addition to social and cognitive deficits, *Arid1b* haploinsufficient mice spend considerably more time grooming than wild type mice, which is considered an appropriate measure of repetitive or stereotyped behaviors in mouse models of ASD^26,27^. *Arid1b* haploinsufficient mice also display significant depression-like behaviors^26^, further replicating human symptoms as depression is a common comorbid condition in individuals with ID and ASD^40^.

With the apparent cortical excitatory/inhibitory imbalance we previously observed in *Arid1b* haploinsufficient mice^26^, we were interested to determine whether ARID1B depletion in cortical or ventral neural progenitors and their progeny contributes to specific behavioral phenotypes individually. We report that homozygous deletion of *Arid1b* in cortical neural progenitors has little to no notable effect on the emotional or social behavior, compared with controls. It does, however, lead to a significant decrease in cognitive function, as evidenced by impaired recognition memory in *Emx-Arid1b* mice (Fig. 6). Conversely, *Arid1b* deletion in ventral neural progenitors leads to considerable social and emotional deficits. We have previously shown that multiple ASD- and ID-like behaviors can be rescued in *Arid1b* haploinsufficient mice by increasing GABA tone using the GABA positive allosteric modulator, clonazepam. This suggests the critical status of ventral neural progenitors and their inhibitory neuronal progeny in *Arid1b* pathology. The current study supports this idea that ARID1B plays an important role in neural behaviors, especially emotional and social activities, by regulating ventral neural progenitors. Future studies will need to examine specific neuronal subpopulations and circuits related to particular behaviors in *Arid1b* mutant mice to provide a better understanding of the mechanistic link between ARID1B and behavior.

Taken together, we conclude that ARID1B plays distinct, yet overlapping roles in cortical and ventral neural progenitors. As multiple BAF subunits have been linked to the regulation of cell proliferation and survival^43^, the function of BAF complexes can be both cell-type and subunit-composition dependent^37,43–48,50,52,56,59,63^. It is likely that ARID1B indeed fulfills different roles in divergent cell populations at different times. Our study reiterates that mutations to a single gene can have a broad impact on multiple cell types and regulate diverse functions.

## Methods

### Generation of conditional *Arid1b* knockout mice

Knockout-first *Arid1b* mutant mice were developed using a C57BL6 background (Jackson Laboratory, #000058) in the Mouse Genome Engineering Core Facility at the University of Nebraska Medical Center as described previously^26^. Homozygous floxed mice were crossed with appropriate Cre drivers (Jackson Laboratory, #005628, #023724 and #008661) for tissue-specific *Arid1b* deletion. All mice were housed in cages with 12:12-h light:dark cycles. No more than five mice were housed per cage. Mice were handled according to a protocol approved by the Institutional Animal Care and Use Committees at the University Nebraska Medical Center and Kent State University. All experiments were performed in accordance with relevant guidelines and regulations.

### Immunostaining

Immunostaining of brain sections or dissociated cells was performed as described previously^26,35,64^. Primary antibodies used were mouse anti-ARID1B (Abcam, ab57461; Abnova, H00057492-M02), rabbit anti-cleaved caspase-3 (Cell Signaling Technology, #9664), mouse anti-BrdU (BD Biosciences, #555627), rabbit anti-p-histone-H3 (Cell Signaling Technology, #9701), rabbit anti-Ki67 (Cell Signaling, #9129), rabbit anti-β-catenin (Cell Signaling, #8480), chicken anti-GFP (Invitrogen, A10262), mouse anti-Parvalbumin (Sigma-Aldrich, MAB1572), rabbit anti-Cux1 (Sigma-Aldrich, SAB2105137), and rabbit anti-Tbr2 (Sigma-Aldrich, AB2283). Appropriate secondary antibodies conjugated with Alexa Fluor dyes (Invitrogen, A32731, A32727, A32723, A32732) were used to detect primary antibodies. DAPI (Sigma-Aldrich, D9542) was used to stain nuclei. No antigen retrieval was performed in this study.

### Stereology

For quantifying numbers of cells, images of 10 different 50 µm brain sections were taken at periodic distances along the rostrocaudal axis with a Zeiss LSM710 confocal microscope as described previously^26,35,65^. *N* values for each experiment are described in figure legends. Where indicated, only cells within certain regions of interest (2.5 mm^2^ white boxes within the VZ of the dorsal telencephalon or MGE) were analyzed in each section. Mouse cultured neurons were also assessed with this microscope. Cell numbers are described in figure legends. Images were analyzed using ZEN (Zeiss) and ImageJ (NIH). The calculated values were averaged, and some results were given as a percentage of DAPI labeled nuclei in the analyzed region.

### BrdU administration and cell cycle analysis

For proliferation assays, BrdU administration and analysis of cell cycle speed and re-entry were performed as described previously^35,36^. Intraperitoneal injection of BrdU (20 mg per kg body weight, dissolved in 0.9% saline) into pregnant mice at E14.5 was performed 30 min prior to sacrifice. Sections from these mice were also used for quantifying overall Ki67 and BrdU labeling in Figs. 1 and 2. For the analysis of cell cycle re-entry, control and mutant mice were exposed to BrdU for 24 h. Brain slices were then immunostained with antibodies to BrdU and Ki67. These sections were also used to evaluate Ki67 expression in Figs. 1 and 2, but not for evaluating BrdU. The ratio of cells labeled with BrdU and Ki67 to total cells that incorporated BrdU was determined. For the analysis of cell cycle length, the ratio of progenitor cells positive for Ki67 and BrdU to the total Ki67 labeled cells was assessed after a 1 h BrdU pulse.

### Cell culture

MGE cells were isolated and cultured from E12.5 to E14.5 mice as described previously^26,66^. Meninges were removed and MGE cells were dissociated with trituration after trypsin/EDTA treatment. The cells were plated onto poly-d-lysine/laminin-coated coverslips and cultured in a medium containing Neurobasal medium (Invitrogen), 2 mM glutamine, 2% (v/v) B27 supplement (Invitrogen), 1% (v/v) N2 supplement (Invitrogen), and 50 U/mL penicillin/streptomycin (Invitrogen). Cells were cultured in these conditions for 48 h and then fixed with 4% paraformaldehyde prior to immunostaining.

### Colocalization

Object-corrected fluorescence colocalization was performed as described by Moser et al.^39^. Briefly, 10 sequential, individual Z stack images, spaced approximately 4 μm apart, were captured using a Zeiss LSM710 confocal microscope and analyzed for colocalization between DAPI and β-catenin using ImageJ (NIH) software with the macro described in the above reference^39^. The mean corrected Pearson’s coefficient for each sample was recorded and the mean of all samples for each genotype is reported in the results.

### Behavioral assays

All behavioral assays were performed as described previously^26,67,68^. Behaviors were assessed during the light cycle. Weight, activity, and feeding as health measures were considered before experiments. 3- to 4-month-old male and female mice were used for most behavioral assays. For social behavior assays, we used only male mice as sexual interactions between males and females and estrous cycle timing may interfere with accurate interpretation of social behavior. All behavioral assays were done blind to genotypes, with age-matched littermates of mice.

### Novel-object recognition test

The novel-object recognition test was performed as previously described^26,68^. A test mouse was first habituated to an open field arena (35 × 42 cm) for 5 min. Following habituation, the test mouse was removed from the arena and two identical objects with size (10.5 × 4.5 × 2.5 cm) were placed in opposite corners of the arena, 7 cm from the side walls. The test mouse was then reintroduced into the center of the arena and allowed to explore the field, including the two novel objects, for 10 min. After 6 h, one object was replaced with another novel object, which was of similar size but different shape and color than the previous object. The same test mouse was placed in the arena to explore the arena and the two objects. The movement of the mouse was recorded by a camera for 10 min and further analyzed by EthoVision XT 7 video tracking software (Noldus).

### Three-chamber test for social interaction and novelty behavior

Social behavior was evaluated as described previously^26,67,68^. A rectangular, transparent Plexiglas box divided by walls into three equal-sized compartments (Ugobasile) was used. Rectangular holes in the Plexiglas walls provide access between the chambers. For sociability testing, a test mouse was moved to the center chamber (chamber 2) with the entrances to the two connecting chambers blocked. A stimulus mouse (unfamiliar mouse) designated as ‘stranger I’ was placed in a wire enclosure in chamber 1. Then the openings to the flanking two chambers (1 and 3) were opened and the test mouse was allowed to explore the entire apparatus for 10 min. For the social novelty test, the stranger I mouse was randomly placed in one of the enclosures, while the test mouse had the choice of whether to investigate the stranger I mouse or a new novel mouse, designated ‘stranger II’. This second novel mouse was taken from a different home cage and placed into the remaining empty wire enclosure. Time spent sniffing each partner by the test mouse was recorded for 10 min in both sociability and social novelty behavior tests. All apparatus chambers were cleaned with water and dried between trials. At the end of each test day, the apparatus was sprayed with 70% ethanol and wiped clean.

### Grooming

Grooming was assessed as previously described^26,68^. A mouse was placed in a clear plastic cage (17 × 32 × 14 cm). The mouse was allowed to freely explore the cage for the entirety of the test. The first 10 min served as a habituation period. The movement of the mouse was recorded by a camera for 30 min. Recorded grooming behaviors included head washing, body grooming, genital/tail grooming, and paw and leg licking.

### Elevated plus maze test

The elevated plus maze test was performed as previously described^26,67,68^. The apparatus (EB Instrument) includes two open arms (35 × 5 cm), two enclosed arms (35 × 5 × 15 cm), and a central platform (5 × 5 cm). The entire apparatus was elevated 45 cm above the floor. A mouse was placed on the central platform, facing the open arms, and allowed to roam freely for 5 min. The number of entries into and the time spent in open and closed arms were recorded.

### Open field test

Open field activity was examined as previously described^26,68^. A mouse was placed near the wall-side of a 35 × 42 cm open field arena, and the movement of the mouse was recorded by a camera for 5 min. The recorded video file was further analyzed using EthoVision XT 7.0 (Noldus). The number of entries into and the overall time spent in the center of the arena (15 × 15 cm imaginary square) were measured. The open field arena was cleaned with 70% ethanol and allowed to dry between each trial.

### Forced swimming test

Forced swimming test was performed as previously described^26,68^. A mouse was placed individually into a glass cylinder (20 cm height, 17 cm diameter) filled with water to a depth of 10 cm at 25 °C. After 5 min, the animals were removed from the water, dried, and returned to their home cages. They were again placed in the cylinder 24 h later, and after the initial 1 min acclimatization period, the total duration of immobility was measured for 5 min. Motionless floating was considered immobile behavior.

### Tail suspension test

Tail suspension behavior was evaluated as described previously^26,68^. A mouse was suspended from the hook of a tail suspension test box, 60 cm above the surface of a table, using adhesive tape placed 1 cm from the tip of the tail. After 1 min acclimatization, immobility duration was recorded by a camera for 5 min. Mice were considered immobile only when they hung passively and were completely motionless.

### Statistical analysis

Statistical analysis was performed as described previously^26,68^. Normal distribution was tested using the Kolmogorov–Smirnov test and variance was compared. Unless otherwise stated, statistical significance was determined using two-tailed, unpaired Student’s *t* tests for two-population comparisons or one-way ANOVA followed by Bonferroni’s post hoc test for multiple comparisons. Data were analyzed using GraphPad Prism and presented as means ± S.E.M. *P* values for each comparison are described in the results or figure legends. To determine and confirm sample sizes (*n*), we performed power analysis and/or surveyed the literature. Each experiment in this study was performed blind and randomized. Animals were assigned randomly to the various experimental groups, and data were collected and processed randomly. The allocation, treatment, and handling of animals was the same across study groups. Control animals were selected from the same litter as the test group. The individuals conducting the experiments were blinded to group allocation and allocation sequence. Exclusion criteria for mice were based on abnormal health conditions, including weights below 15 g at 6 weeks of age and noticeably reduced activity or feeding. Statistical data and *n* values for all behavioral assays are described in the figure legends.

This study was carried out in compliance with the ARRIVE guidelines (http://www.nc3rs.org.uk/page.asp?id=1357).

## Supplementary Information

Supplementary Information.

## References

[1] Perou R (2013). Mental health surveillance among children—United States, 2005–2011. MMWR Suppl..

[2] Srivastava AK, Schwartz CE (2014). Intellectual disability and autism spectrum disorders: Causal genes and molecular mechanisms. Neurosci. Biobehav. Rev..

[3] Collins RT, Furukawa T, Tanese N, Treisman JE (1999). Osa associates with the Brahma chromatin remodeling complex and promotes the activation of some target genes. EMBO J..

[4] Wang X (2004). Two related ARID family proteins are alternative subunits of human SWI/SNF complexes. Biochem. J..

[5] Moffat JJ (2019). The role of ARID1B, a BAF chromatin remodeling complex subunit, in neural development and behavior. Prog. Neuropsychopharmacol. Biol. Psychiatry.

[6] Santen GW (2012). Mutations in SWI/SNF chromatin remodeling complex gene ARID1B cause Coffin–Siris syndrome. Nat. Genet..

[7] Tsurusaki Y (2012). Mutations affecting components of the SWI/SNF complex cause Coffin–Siris syndrome. Nat. Genet..

[8] Mariani J (2015). FOXG1-dependent dysregulation of GABA/glutamate neuron differentiation in autism spectrum disorders. Cell.

[9] Chanas-Sacre G, Rogister B, Moonen G, Leprince P (2000). Radial glia phenotype: Origin, regulation, and transdifferentiation. J. Neurosci. Res..

[10] Noctor SC, Flint AC, Weissman TA, Dammerman RS, Kriegstein AR (2001). Neurons derived from radial glial cells establish radial units in neocortex. Nature.

[11] Hartfuss E, Galli R, Heins N, Gotz M (2001). Characterization of CNS precursor subtypes and radial glia. Dev. Biol..

[12] Rakic P (1972). Mode of cell migration to the superficial layers of fetal monkey neocortex. J. Comp. Neurol..

[13] Stiles J, Jernigan TL (2010). The basics of brain development. Neuropsychol. Rev..

[14] Sultan KT, Brown KN, Shi SH (2013). Production and organization of neocortical interneurons. Front. Cell. Neurosci..

[15] Anderson SA, Marin O, Horn C, Jennings K, Rubenstein JL (2001). Distinct cortical migrations from the medial and lateral ganglionic eminences. Development.

[16] Molyneaux BJ, Arlotta P, Menezes JR, Macklis JD (2007). Neuronal subtype specification in the cerebral cortex. Nat. Rev. Neurosci..

[17] Gatto CL, Broadie K (2010). Genetic controls balancing excitatory and inhibitory synaptogenesis in neurodevelopmental disorder models. Front. Synaptic Neurosci..

[18] Nelson SB, Valakh V (2015). Excitatory/inhibitory balance and circuit homeostasis in autism spectrum disorders. Neuron.

[19] Rubenstein JL, Merzenich MM (2003). Model of autism: Increased ratio of excitation/inhibition in key neural systems. Genes Brain Behav..

[20] Cellot G, Cherubini E (2014). GABAergic signaling as therapeutic target for autism spectrum disorders. Front. Pediatr..

[21] Nagl NG, Wang X, Patsialou A, Van Scoy M, Moran E (2007). Distinct mammalian SWI/SNF chromatin remodeling complexes with opposing roles in cell-cycle control. EMBO J..

[22] Taniguchi H (2011). A resource of Cre driver lines for genetic targeting of GABAergic neurons in cerebral cortex. Neuron.

[23] Gorski JA (2002). Cortical excitatory neurons and glia, but not GABAergic neurons, are produced in the Emx1-expressing lineage. J. Neurosci..

[24] Monory K (2006). The endocannabinoid system controls key epileptogenic circuits in the hippocampus. Neuron.

[25] Zerucha T (2000). A highly conserved enhancer in the Dlx5/Dlx6 intergenic region is the site of cross-regulatory interactions between Dlx genes in the embryonic forebrain. J. Neurosci..

[26] Jung EM (2017). Arid1b haploinsufficiency disrupts cortical interneuron development and mouse behavior. Nat. Neurosci..

[27] Celen C (2017). Arid1b haploinsufficient mice reveal neuropsychiatric phenotypes and reversible causes of growth impairment. Elife.

[28] Shibutani M (2017). Arid1b haploinsufficiency causes abnormal brain gene expression and autism-related behaviors in mice. Int. J. Mol. Sci..

[29] Hans F, Dimitrov S (2001). Histone H3 phosphorylation and cell division. Oncogene.

[30] Scholzen T, Gerdes J (2000). The Ki-67 protein: From the known and the unknown. J Cell Physiol.

[31] Cuylen S (2016). Ki-67 acts as a biological surfactant to disperse mitotic chromosomes. Nature.

[32] Gratzner HG (1982). Monoclonal antibody to 5-bromo- and 5-iododeoxyuridine: A new reagent for detection of DNA replication. Science.

[33] Nowakowski RS, Lewin SB, Miller MW (1989). Bromodeoxyuridine immunohistochemical determination of the lengths of the cell cycle and the DNA-synthetic phase for an anatomically defined population. J. Neurocytol..

[34] Wojtowicz JM, Kee N (2006). BrdU assay for neurogenesis in rodents. Nat. Protoc..

[35] Ka M, Smith AL, Kim WY (2017). MTOR controls genesis and autophagy of GABAergic interneurons during brain development. Autophagy.

[36] Kee N, Sivalingam S, Boonstra R, Wojtowicz JM (2002). The utility of Ki-67 and BrdU as proliferative markers of adult neurogenesis. J. Neurosci. Methods.

[37] Vasileiou G (2015). Chromatin-remodeling-factor ARID1B represses Wnt/beta-catenin signaling. Am. J. Hum. Genet..

[38] Wu J (2016). Insertional mutagenesis identifies a STAT3/Arid1b/beta-catenin pathway driving neurofibroma initiation. Cell. Rep..

[39] Moser B, Hochreiter B, Herbst R, Schmid JA (2017). Fluorescence colocalization microscopy analysis can be improved by combining object-recognition with pixel-intensity-correlation. Biotechnol. J..

[40] Wing L, Gould J (1979). Severe impairments of social interaction and associated abnormalities in children: Epidemiology and classification. J. Autism. Dev. Disord..

[41] Gillott A, Furniss F, Walter A (2001). Anxiety in high-functioning children with autism. Autism.

[42] van Steensel FJA, Heeman EJ (2017). Anxiety levels in children with autism spectrum disorder: A meta-analysis. J. Child. Fam. Stud..

[43] Son EY, Crabtree GR (2014). The role of BAF (mSWI/SNF) complexes in mammalian neural development. Am. J. Med. Genet. C Semin. Med. Genet..

[44] Battaglioli E (2002). REST repression of neuronal genes requires components of the hSWI.SNF complex. J. Biol. Chem..

[45] Tuoc TC (2013). Chromatin regulation by BAF170 controls cerebral cortical size and thickness. Dev. Cell..

[46] Olave I, Wang W, Xue Y, Kuo A, Crabtree GR (2002). Identification of a polymorphic, neuron-specific chromatin remodeling complex. Genes Dev..

[47] Wu JI (2007). Regulation of dendritic development by neuron-specific chromatin remodeling complexes. Neuron.

[48] Lessard J (2007). An essential switch in subunit composition of a chromatin remodeling complex during neural development. Neuron.

[49] Chiba H, Muramatsu M, Nomoto A, Kato H (1994). Two human homologues of Saccharomyces cerevisiae SWI2/SNF2 and Drosophila brahma are transcriptional coactivators cooperating with the estrogen receptor and the retinoic acid receptor. Nucleic Acids Res.

[50] Hodges C, Kirkland JG, Crabtree GR (2016). The many roles of BAF (mSWI/SNF) and PBAF complexes in cancer. Cold Spring Harb. Perspect. Med..

[51] Hurlstone AF, Olave IA, Barker N, van Noort M, Clevers H (2002). Cloning and characterization of hELD/OSA1, a novel BRG1 interacting protein. Biochem. J..

[52] Inoue H (2002). Largest subunits of the human SWI/SNF chromatin-remodeling complex promote transcriptional activation by steroid hormone receptors. J. Biol. Chem..

[53] Barker N (2001). The chromatin remodelling factor Brg-1 interacts with beta-catenin to promote target gene activation. EMBO J..

[54] Liu X (2020). De Novo ARID1B mutations cause growth delay associated with aberrant Wnt/beta-catenin signaling. Hum. Mutat..

[55] de la Serna IL, Carlson KA, Imbalzano AN (2001). Mammalian SWI/SNF complexes promote MyoD-mediated muscle differentiation. Nat. Genet..

[56] Lickert H (2004). Baf60c is essential for function of BAF chromatin remodelling complexes in heart development. Nature.

[57] Ohkawa Y, Marfella CG, Imbalzano AN (2006). Skeletal muscle specification by myogenin and Mef2D via the SWI/SNF ATPase Brg1. EMBO J..

[58] Cvekl A, Duncan MK (2007). Genetic and epigenetic mechanisms of gene regulation during lens development. Prog. Retin. Eye Res..

[59] Li W (2013). Brg1 governs distinct pathways to direct multiple aspects of mammalian neural crest cell development. Proc. Natl. Acad. Sci. USA.

[60] Xiong Y (2013). Brg1 governs a positive feedback circuit in the hair follicle for tissue regeneration and repair. Dev. Cell..

[61] Yu Y (2015). De novo mutations in ARID1B associated with both syndromic and non-syndromic short stature. BMC Genom..

[62] Gulacsi AA, Anderson SA (2008). Beta-catenin-mediated Wnt signaling regulates neurogenesis in the ventral telencephalon. Nat. Neurosci..

[63] Narayanan R (2015). Loss of BAF (mSWI/SNF) complexes causes global transcriptional and chromatin state changes in forebrain development. Cell. Rep..

[64] Ka M, Kook YH, Liao K, Buch S, Kim WY (2016). Transactivation of TrkB by Sigma-1 receptor mediates cocaine-induced changes in dendritic spine density and morphology in hippocampal and cortical neurons. Cell Death Dis..

[65] Ka M, Moffat JJ, Kim WY (2017). MACF1 controls migration and positioning of cortical GABAergic interneurons in mice. Cereb. Cortex.

[66] Ka M, Kim WY (2018). ANKRD11 associated with intellectual disability and autism regulates dendrite differentiation via the BDNF/TrkB signaling pathway. Neurobiol. Dis..

[67] Smith AL, Jung EM, Jeon BT, Kim WY (2020). Arid1b haploinsufficiency in parvalbumin- or somatostatin-expressing interneurons leads to distinct ASD-like and ID-like behavior. Sci. Rep..

[68] Jung EM, Ka M, Kim WY (2016). Loss of GSK-3 causes abnormal astrogenesis and behavior in mice. Mol. Neurobiol..

