# Supplementary material for: Differential roles of ARID1B in excitatory and inhibitory neural progenitors in the developing cortex
Source: Sci Rep. 2021 Feb 16;11:3856. doi: 10.1038/s41598-021-82974-y (PMC7886865; doi:10.1038/s41598-021-82974-y)

TITLE: Differential roles of ARID1B in excitatory and inhibitory neural progenitors in the developing cortex

Jeffrey J. Moffat[1#], Eui-Man Jung[2], Minhan Ka[3], Byeong Tak Jeon[4], Hyunkyoung Lee[4], and Woo-Yang Kim[4]*

[1]Developmental Neuroscience, Munroe-Meyer Institute, University of Nebraska Medical Center, Omaha, NE 68198

[2]Laboratory of Veterinary Biochemistry and Molecular Biology, Chungbuk National University, Cheongju, Chungbuk 28644, Republic of Korea

[3]Research Center for Substance Abuse Pharmacology, Korea Institute of Toxicology, Daejeon 34114, Republic of Korea

[4]Department of Biological Sciences, Kent State University, Kent, OH 44242, USA

[#]Current Address: Department of Neurology, University of California San Francisco, San Francisco, CA 94153

* Corresponding author

Supplementary Figure 1. *Arid1b* knockout in *Emx-Arid1b* and *Dlx-Arid1b* brains

(A) Western blots confirmed conditional *Arid1b* knockout in the cerebral cortex and MGE of *Emx-Arid1b* and *Dlx-Arid1b* mice, respectively. (B) Quantification of (A). N=4 mice for each genotype. Statistical significance was determined by two-tailed Student's t-test. Error bars show SEM.

Supplementary Figure 2. Background cell numbers in tissue samples examined

(A) and (B) The numbers of DAPI-positive cells did not show significant changes in either *Emx-Arid1b* and *Dlx-Arid1b* samples. Statistical significance was determined by two-tailed Student's t-test. Error bars show SEM.

Supplementary Figure 3. The number of mature neurons in the cerebral cortex of *Dlx-Arid1b* mice

(A) Cortical sections of P30 control and *Dlx-Arid1b* mice were immunostained with an anti-NeuN antibody. (B) Quantification of (A). N=15 mice for each condition. Statistical significance was determined by two-tailed Student's t-test. Error bars show SEM.

Supplementary Figure 1

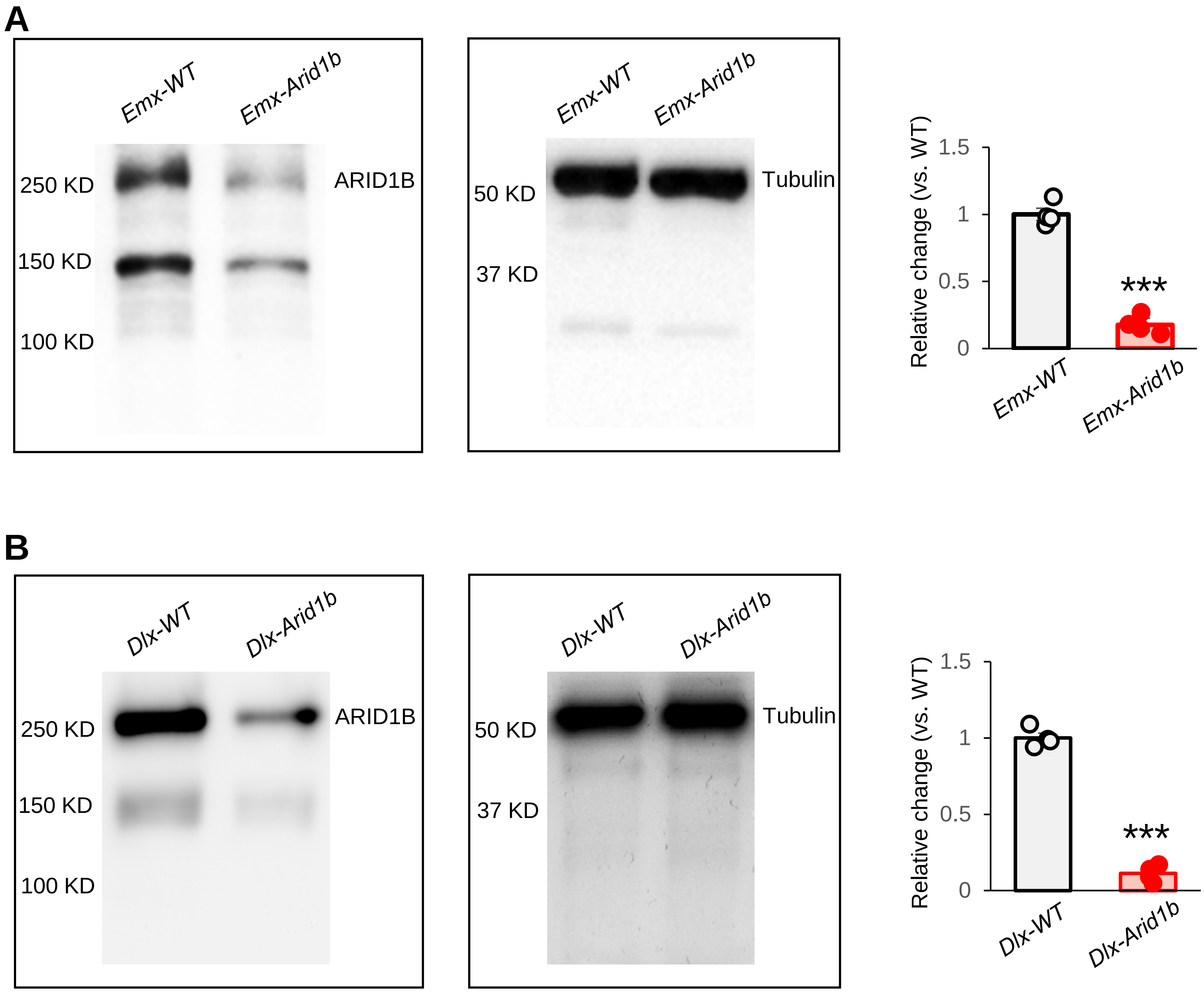

Supplementary Figure 2

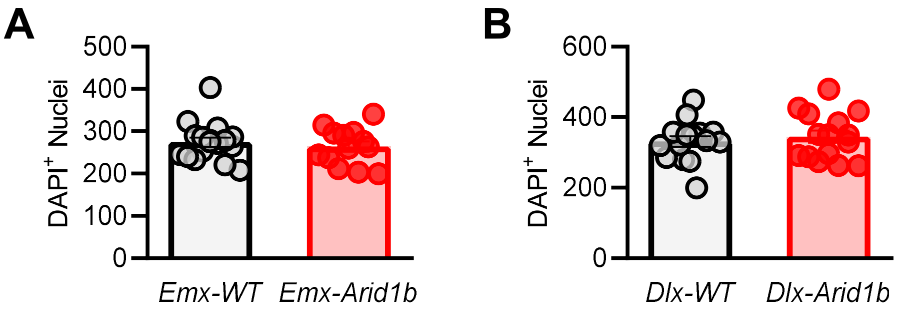

Supplementary Figure 3

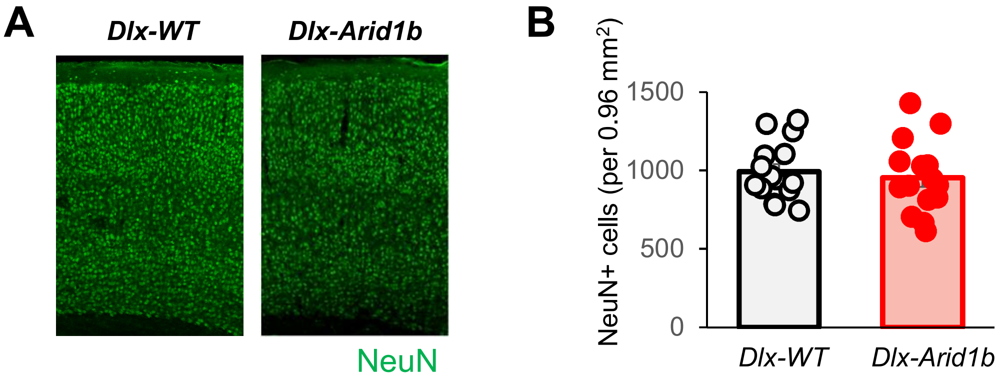

Supplement: Supplementary file 1 — Supplementary Information. [file 41598_2021_82974_MOESM1_ESM.pdf]
